# Prime-boost regimen potency and efficacy with alphavirus replicons (SIV antigen) in non-human primates challenged with low-dose intra-rectal SIVsmE660

**DOI:** 10.1186/1742-4690-9-S2-O8

**Published:** 2012-09-13

**Authors:** K Banerjee, S Balsitis, T Jamil, C Jones, A Dey, J Flandez, L Brito, Y Cu, C Beard, S Santra, R Pal, N Miller, NM Valiante, P Mason, SW Barnett, GR Otten

**Affiliations:** 1 Novartis Vaccines and Diagnostics, Cambridge, MA, USA; 2 Beth Israel Deaconess Medical Center, Boston, MA, USA; 3 Advanced Biosciences Laboratories, Kensington, MD, USA; 4 Division of AIDS, NIAID, MD, USA

## Background

Self-amplifying RNAs (replicons) of positive-strand viruses such as alphaviruses are potentially safe and useful vectors for delivering vaccine antigens. Recombinant alphavirus replicon particles (VRP), carrying the self-amplifying RNA, protects rhesus macaques against SHIVSF162P4 challenge when used in a prime-boost regimen.

## Methods

Novartis VRPs are being further tested using a current state-of-the art physiologically relevant low-dose SIV virus swarm challenge. To meet the need for the large numbers of VRP an alphavirus packaging cell line (PCL) was used for VRP production. We manufactured, characterized, stability and small animal potency tested VRPs expressing SIVmac239 envelope (env) and gag/pol fusion proteins (VRP Env, VRP Gag/pol, respectively) for a large macaque vaccine study. Macaques were co-immunized with both VRPs thrice followed by two boosts with an MF59-adjuvanted CHO cell-derived SIVmac239 trimeric env protein.

## Results

Here we show that three VRP priming immunizations induce both env- and gag-specific IgG and T-cell responses, robustly. Binding env-specific IgG titers were demonstrable in 100% of animals with titers ranging from ~10000-400000. T-cell responses to env and gag developed in 80% of macaques (env-specific range ~100-1200, gag-specific range ~100-700 SFC/106 PBMCs) when assayed using an IFNγ T-cell ELISpot. The MF59-adjuvanted Env protein by itself was also robustly immunogenic with Env-specific IgG titers and T-cell responses ranging from ~100000-1700000 (100% response) and ~100-1000 SFC/106 PBMCs (90% response), respectively. No adverse events were reported upon immunization of either the VRP or the MF59-adjuvanted env vaccines.

## Conclusion

We provide further details on the currently ongoing efficacy evaluations of these safe and immunogenic vaccines in a prime-boost regimen using repeated low-dose heterologous SIVsmE660 intra-rectal challenges. NIH Grant N01-AI-50007.

